# Mini Review: Comparison of Bio-Inspired Adhesive Feet of Climbing Robots on Smooth Vertical Surfaces

**DOI:** 10.3389/fbioe.2021.765718

**Published:** 2021-09-30

**Authors:** Pongsiri Borijindakul, Aihong Ji, Zhendong Dai, Stanislav N. Gorb, Poramate Manoonpong

**Affiliations:** ^1^ Institute of Bio-inspired Structure and Surface Engineering, College of Mechanical and Electrical Engineering, Nanjing University of Aeronautics and Astronautics, Nanjing, China; ^2^ Department of Functional Morphology and Biomechanics, Zoological Institute, Kiel University, Kiel, Germany; ^3^ Embodied Artificial Intelligence and Neurorobotics Laboratory, SDU Biorobotics, The Mærsk Mc-Kinney Møller Institute, University of Southern Denmark, Odense M, Denmark

**Keywords:** bio-inspired climbing robots, smooth vertical surfaces, adhesive foot, spatula-shaped, mushroom-shaped

## Abstract

Developing climbing robots for smooth vertical surfaces (e.g., glass) is one of the most challenging problems in robotics. Here, the adequate functioning of an adhesive foot is an essential factor for successful locomotion performance. Among the various technologies (such as dry adhesion, wet adhesion, magnetic adhesion, and pneumatic adhesion), bio-inspired dry adhesion has been actively studied and successfully applied to climbing robots. Thus, this review focuses on the characteristics of two different types of foot microstructures, namely spatula-shaped and mushroom-shaped, capable of generating such adhesion. These are the most used types of foot microstructures in climbing robots for smooth vertical surfaces. Moreover, this review shows that the spatula-shaped feet are particularly suitable for massive and one-directional climbing robots, whereas mushroom-shaped feet are primarily suitable for light and all-directional climbing robots. Consequently, this study can guide roboticists in selecting the right adhesive foot to achieve the best climbing ability for future robot developments.

## 1 Introduction

**FIGURE 1:**
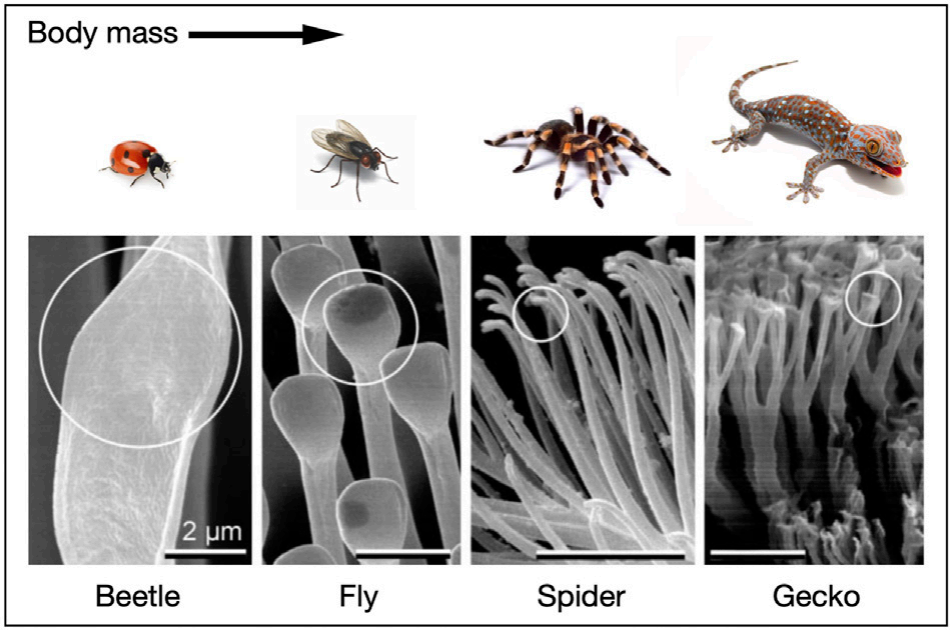
Terminal elements of the hairy attachment pads of a beetle, fly, spider, and gecko. The heavier animals show finer spatula structures [courtesy of Nate Abbott for the image of the gecko and modified from Arzt et al. (2003) for images fo the spatula-like terminal elements].

Bio-inspired climbing robots have been widely studied over the past 10 years. [Daltorio et al. (2005); Unver et al. (2005); Daltorio et al. (2006); Daltorio et al. (2007a); Daltorio et al. (2007b); Santos et al. (2008); Sameoto et al. (2008); Menon et al. (2008); Daltorio et al. (2008); Wile et al. (2008b); Wile et al. (2008a); Daltorio et al. (2009); Li et al. (2012); Seitz et al. (2014); Tavakoli and Viegas (2015); Elbadawi et al. (2018); Schiller et al. (2019); Srisuchinnawong et al. (2019)]. An essential factor for climbing robots is adhesion [Silva et al. (2008)], a fundamental phenomenon in nature. Some animals can walk or climb vertical terrains and ceilings using adhesive feet, such as ladybugs, flies, spiders, and geckos. Biological adhesion can be classified into two types: wet and dry. Insects produce a liquid secretion from their feet to adhere to a substrate [Peisker and Gorb (2012); Kovalev et al. (2013); Peisker et al. (2014); Gilet et al. (2018)]. Gastropods adhere to a surface by generating a thin layer of pedal mucus on surfaces [Denny (1980a); Denny (1980b); Denny (1981)]. Harvestmen use viscoelastic fluids to capture small arthropods such as springtails [Wolff et al. (2014); Wolff et al. (2016)]. In particular, the viscoelastic fluids provide wet adhesion. By contrast, the dry adhesion in spiders and geckos is achieved by deformable setae with substrates, which generates an intermolecular adhesion force between the setae and surfaces [Arzt et al. (2003); Tian et al. (2006); Bhushan (2008); Autumn et al. (2014)]. In addition to deformable setae, Autumn et al. (2002) demonstrated that van der Waals forces are also responsible for the dry adhesion of gecko setae. The pads of beetles and flies are divided into setae with flat tips that resemble mushroom shapes or spatulate shapes. The pads of geckos and spiders consist of lamellae, subdivided into setae branches. Furthermore, the terminal element branches are widened and flattened at the tip, making them look like spatula shapes [Peressadko and Gorb (2004); Wolff and Gorb (2012a); Wolff and Gorb (2012b); Wolff and Gorb (2012c); Autumn et al. (2014); Wohlfart et al. (2014); Wolff and Gorb (2015); Frost et al. (2018)]. As the body of the animal increases, the terminal elements of the hairy attachment pads increase in number and density [Arzt et al. (2003); Figure 1]. This allows a more significant number of setae to touch the surface and create a substantial real area of contact. In other words, the real contact area and adhesion strength increase when the dimensions of the terminal elements decrease and their density increase.

Recently, several types of artificial adhesive feet have been developed based on bio-inspiration studies, such as mechanical adhesion (gripping), pneumatic adhesion (suction cups), magnetic adhesion (permanent magnet), and dry adhesion (elastomer adhesive) [Daltorio et al. (2006); Kim et al. (2008); Hu et al. (2009); Seitz et al. (2014); Hawkes et al. (2015); Xu et al. (2016); Jiang and Xu (2018)]; Elbadawi et al. (2018); Chattopadhyay and Ghoshal (2018). In particular, bio-inspired dry adhesion has been actively studied and applied to climbing robots on smooth vertical surfaces. Therefore, in this review, we focus on bio-inspired adhesive feet for such surfaces. In this regard, two different widely used types of adhesive feet exist spatula-shaped feet (Kim et al. (2008)) and mushroom-shaped feet [Peressadko and Gorb (2004); Daltorio et al. (2006)], both approaches are synthetic reversible adhesive tapes. Each method has advantages and disadvantages depending on the robot’s mass, climbing direction, attachment, detachment, and reusability. A review of current approaches of these two types of adhesives could guide future improvement of robots climbing up smooth vertical surfaces.

## 2 Bio-Inspired Adhesive Feet on Climbing Robots

**FIGURE 2:**
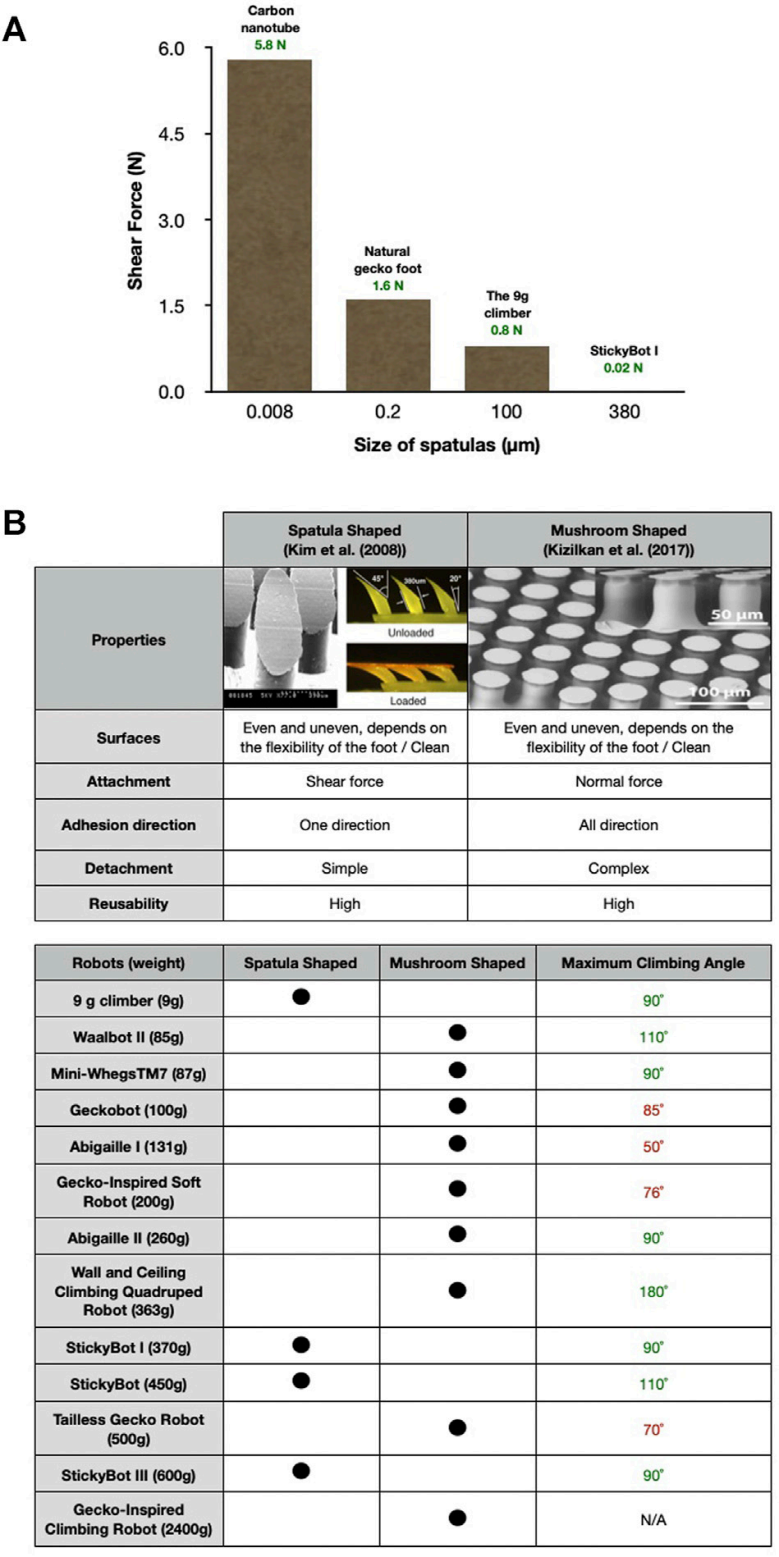
**(A)** The comparison of shear forces between each spatula size of the carbon-nanotube-based synthetic gecko tapes, natural gecko foot, StickyBot I, and the 9 g climber in a 0.16 cm^2^ area [Ge et al. (2007); Santos et al. (2008); Hawkes et al. (2015)] **(B)** Characteristics of adhesive materials with spatula-shaped and mushroom-shaped microstructures.

Synthetic adhesive feet inspired by animals are suitable for climbing robots. Using adhesive feet, robots can walk on steep slopes and vertical surfaces (Figure 2). These surfaces determine the adhesion technologies for the feet, such as magnetic adhesion on ferromagnetic surfaces, mechanical gripping on trees/pipes, and suction/dry adhesion on glass [Chattopadhyay and Ghoshal (2018)]. Spatula-shaped and mushroom-shaped feet are most commonly used in climbing robots, e.g., StickyBot [Santos et al. (2008)], StickyBot I; [Kim et al. (2008)], StickyBot III [Hawkes et al. (2011)], Geckobot [Unver et al. (2005)], Gecko-Inspired Soft Robot [Schiller et al. (2019)], Abigaille I [Menon et al. (2008)], Abigaille II [Li et al. (2012)], Tailless Gecko Robot [Srisuchinnawong et al. (2019)], 9 g climber [Hawkes et al. (2015)], Mini-WhegsTM7 [Daltorio et al. (2006)], Waalbot II [Murphy et al. (2011)], Wall and Ceiling Climbing Quadruped Robot [Ko et al. (2017)], and Gecko-Inspired Climbing Robot [Shao et al. (2020)]. Thus, this review mainly focuses on bio-inspired adhesive feet for smooth vertical surfaces such as glass.

### 2.1 Spatula-Shaped

A synthetic dry adhesive inspired by geckoes, directional polymer stalks (DPSs), was designed and manufactured to create a directional adhesive similar to that in gecko feet. DPSs are made from polyurethane (Innovative Polymers, IE-20 AH polyurethane, 20 Shore-A hardness, E ≈ 300 kPa). DPSs comprise an array of micro-spatula-shaped polymer features [Kim et al. (2008); Figure 2B]. The spatula-shaped feet are soft materials that are marginally sticky. Moreover, geometric properties were determined empirically, drawing considering the shapes of gecko setae. Moreover, the DPSs make contact without a high normal preload. The sharp and thin tip shape of the DPS is designed to create a softer sufficient stiffness when pulled parallel to the terrains [Kim et al. (2008)]. The adhesion disappears by pushing or lifting against the shear force. The adhesion force depends on the polymer type, adequate direction, and size of the spatulas. If we consider the spatula size in these robots, Stickybot I have a larger size (380 μm) than the 9 g climber (100 μm) [Kim et al. (2008); Hawkes et al. (2015)]. Stickybot I could not lift over 100% of its body weight [Hawkes et al. (2015)] while, the 9 g climber could climb up a smooth vertical surface while hoisting 1000% of its body weight [Hawkes et al. (2015)]. In contrast to the aforementioned robot spatula-shaped foot, the natural gecko foot has about half a million setae, each of which contains hundreds to thousands of spatulas. The spatulas have an average diameter of 200 nm and an estimated adhesion force of 0.4 μN[(Autumn et al. (2000); Persson and Gorb (2003); Ge et al. (2007)]. Carbon-nanotube-based synthetic gecko tapes consist of thousands of synthetic spatulas with an average diameter of approximately 8 nm which can generate strong adhesion forces. They can adhere more than a natural gecko foot nearly ten times (∼ 100 Ncm−2) [Ge et al. (2007); Qu et al. (2008); Schaber et al. (2015)]. We observe that a smaller size of the spatulas allows for a higher adhesion, as shown in Figure 2A. Furthermore, the feet can be reconditioned by cleaning with soap and water [Kim et al. (2008)]. Therefore, they can be continuously used several times.

### 2.2 Mushroom-Shaped

The mushroom-shaped adhesive microstructure (MSAMS)—inspired by the attachment systems of beetles from the family Chrysomelidae—was made from polyvinyl siloxane (PVS) with a hexagonal patterning height of approximately 100 μm and a base diameter of 60 μm. The adhesive covers almost half of the contact area of the material [Gorb S. et al. (2007); Figure 2B]. The MSAMS makes contact with the preload along the normal force axis and can be detached by the peeling technique at some angles [Shao et al. (2020)]. The adhesion of MSAMS has approximately twice the pull-off force of surface without MSAMS (flat surface) made from the same material, while both were independent of the preload. The MSAMS has more repeatability than flat specimen in the peel strength [Gorb S. et al. (2007)]. A demonstration of its potential showed that a 20 cm × 20 cm tape supported a weight of approximately 70 kg [Heepe et al. (2012)]. The MSAMS also provides adhesion underwater [Heepe et al. (2012); Heepe and Gorb (2014); Ko et al. (2017)] and has no directional adhesion [Murphy et al. (2011); Seibel et al., 2020)]; therefore, it is one of the most used methods for smooth vertical surface climbing robots. Furthermore, the tape can be reconditioned by cleaning with soap and water [Gorb S. N. et al. (2007)]. Therefore, it can be continuously used multiple times.

## 3 Discussion

Spatula- and mushroom-shaped microstructures can be used on both even and uneven, clean surfaces because of their flexibility [Kim et al. (2008); Gorb S. et al. (2007)]. The attachment system of spatula-shaped feet is very simple. They require only shear force to attach to surfaces [Hawkes et al. (2015)]. By contrast, mushroom-shaped feet require a slight initial normal force to attach to surfaces [Kim et al. (2008); Shao et al. (2020)]. The detachment mechanism of the spatula-shaped microstructure is also straightforward. The adhesion force disappears when the feet are pushed or lifted against the shear force. The system in mushroom-shaped feet is more complicated than the spatula-shaped. The former requires a specific peeling angle for detachment [Shao et al. (2020)].

Furthermore, the climbing direction of the spatula-shaped feet is limited to one-directional adhesion; therefore, robots can climb up to 90 and 110° depending on the foot orientation [Santos et al. (2008)]. In contrast, mushroom-shaped feet provide all-directional adhesion; hence, robots can climb on the ceiling [Murphy et al. (2011); Ko et al. (2017)]. The reusability of the spatula-shaped feet is impressive. Some Stickybot feet with a spatula-shaped structure have been continuously used for over 6 months without significant performance loss [Kim et al. (2008)]. In addition, cleaning the feet with soap and water before use can recondition the adhesion ability [Kim et al. (2008)]. However, mushroom-shaped feet are relatively less reusable. The detachment system damages the structure from time to time [Gorb S. et al. (2007)]. However, cleaning the feet before use could also recondition the adhesion ability (Figure 2B).

## 4 Conclusion

Both spatula-shaped feet and mushroom-shaped feet have advantages and disadvantages. The substrate surface, robot mass, climbing direction, adhesion force, and reusability are the primary factors to consider when choosing adequate feet. Spatula-shaped feet are suitable for heavy climbing robots as they provide an adhesion force on a shear force axis. In contrast, mushroom-shaped feet provide an adhesion force on the normal force axis. For instance, the 9 g climber could climb while hoisting 1 kg up on a smooth vertical surface [Hawkes et al. (2015)], while the maximum slope climbing angle of the tailless gecko robot (500 g) was 70° [Srisuchinnawong et al. (2019)]. Moreover, the mushroom-shaped feet could climb in all directions because the foot structure provides all-directional adhesion [Murphy et al. (2011); Li et al. (2012)]. However, spatula-shaped feet provide only one direction of adhesion. If robots intend to adhere to overhanging surfaces or ceilings, they should have the ability to change their foot orientation [Santos et al. (2008)]. Finally, reusability is also essential. In this regard, spatula-shaped feet have apparently higher reusability [Gorb S. et al. (2007); Kim et al. (2008)]. However, cleaning the feet before use can restore the adhesion ability of both types of microstructure.
